# Patient-reported outcome measures and clinical outcomes following peri-implant vestibuloplasty with a free gingival graft versus xenogeneic collagen matrix: a comparative prospective clinical study

**DOI:** 10.1186/s40729-021-00356-5

**Published:** 2021-08-02

**Authors:** Xiaojiao Fu, Ying Wang, Bo Chen, Jiehua Tian, Ye Lin, Yu Zhang

**Affiliations:** Department of Oral Implantology, Peking University School and Hospital of Stomatology & National Center of Stomatology & National Clinical Research Center for Oral Diseases & National Engineering Laboratory for Digital and Material Technology of Stomatology & Beijing Key Laboratory of Digital Stomatology & Research Center of Engineering and Technology for Computerized Dentistry Ministry of Health & NMPA Key Laboratory for Dental Materials, No.22 Zhongguancun South Avenue, Haidian District, Beijing, 100081 People’s Republic of China

**Keywords:** Free gingival graft, Xenogeneic collagen matrix, Dental implant, Patient-reported outcome measures

## Abstract

**Background:**

The objective of this study was to compare patient-reported outcome measures (PROMs) and clinical outcomes after augmentation with xenogeneic collagen matrix (XCM) or free gingival graft (FGG) during different postoperative phases.

**Methods:**

Forty-two patients (21 per group) with keratinized mucosa width (KMW) of < 2 mm at buccal implant sites in the posterior mandible were enrolled. All underwent vestibuloplasty and were allocated to either FGG (control) or XCM (test) group. Intraoperative morbidity of pain, stress, nausea, tolerance to time, and acceptance of surgery were evaluated immediately after surgery. The severity and duration of subjective pain, swelling, and bleeding were compared within a 2-week postoperative period. The willingness to retreat and satisfaction were assessed at 6 months. All PROMs were obtained using questionnaires and visual analog scales. The buccal KMW and other peri-implant parameters were also evaluated.

**Results:**

No significant between-group differences were observed in PROMs immediately after surgery, except acceptance of surgery (0, 0–30.0 vs. 30, 0–50.0, *p* = 0.025). At 2 weeks, pain severity (46.7 ± 25.9 vs 61.9 ± 20.2, *p* = 0.040) and duration (5.52 ± 3.57 vs 8.48 ± 2.80, *p* = 0.005) were significantly lower in the test group, and pain perception during speaking and chewing was significantly higher for FGG, with no significant between-group differences in swelling and bleeding. At 6 months, the test group showed a higher willingness to retreat (76% vs 43%, *p* = 0.021); however, satisfaction with treatment outcomes was similar in both groups. At 6 months, the gain of KMW was significantly higher in FGG than in XCM (XCM: 1.57 ± 1.69 mm, FGG: 2.68 ± 1.80 mm, *p* = 0.003). Other peri-implant parameters did not show significant differences.

**Conclusions:**

Within the limitation of the present nonrandomized study, XCM demonstrated more positive PROMs than FGG during different postoperative phases, mainly for less pain perception during the early healing stage, but was inferior to FGG in terms of gain of KMW. For KMW augmentation in the posterior mandible, XCM may be indicated when patients can bear little pain.

**Clinical trial registration:**

ChiCTR1900022575, date of registration: 17/4/2019, retrospectively registered,

## Background

Free gingival graft (FGG) is the most effective technique for increasing the keratinized mucosa width (KMW) around dental implants [1–4]. However, this technique requires harvesting of the autogenous graft from the palate, which leaves an open wound area and is closely associated with increased postoperative morbidity [5, 6]. Excessive pain in the exposed palatal wound has been attributed to secondary intention healing [7, 8].

Xenogeneic collagen matrix (XCM) has been introduced as a substitute material to autogenous graft from the palate. In some suitable cases, XCM has been shown to increase peri-implant keratinized mucosa with similar effectiveness and predictability as FGG [9–11]. Using the XCM avoids the painful tissue harvesting procedures and reduces postoperative morbidity [10–13]. However, studies about using XCM for increasing peri-implant KMW in the mandibular posterior region are rare [4].

Previous studies have mainly compared the clinical effectiveness of FGG and XCM by assessing the increase of KMW [4, 9–11, 14]. However, the decision-making of keratinized mucosa augmentation does not solely depend on the final keratinized mucosa width. Patient-reported outcome measures (PROMs), including satisfaction and patient-reported postoperative discomfort, are other important parameters [15, 16]. To our knowledge, there have been few studies comparing the effect of XCM to that of FGG, particularly from the aspect of PROMs.

There has been a recent increase in clinical studies on PROMs after soft tissue grafting [5, 16–19]. Assessing PROMs at different postoperative phases is beneficial as it allows better informing of patients, setting of patient’s expectations, and provision of the necessary information to facilitate decision-making [17, 20]. Additionally, understanding the patients’ morbidity can allow the adoption of different techniques for reducing patient discomfort and guiding pain management. Therefore, there is a need to evaluate PROMs during different postoperative phases following XCM and FGG. Although these outcomes lack standardized measurement tools, visual analog scales (VAS) and questionnaires are frequently adopted for PROMs assessment [15].

The main objective of this study was to compare FGG with XCM for vestibuloplasty with respect to PROMs during different postoperative phases within a 6-month follow-up period. The secondary objective of this study was to compare clinical outcomes following both procedures in the mandibular posterior region.

## Methods

### Patient groups

This comparative prospective clinical study was approved by the Medical Ethics Committee of Peking University, Health Science Center, School of Stomatology (Approval Number: PKUSSIRB-201839155) and was conducted in accordance with the Helsinki declaration, as revised in 2013. It was conducted at the Department of Oral Implantology of Peking University School and Hospital of Stomatology from November 2018 to December 2019. All the patients gave their informed consent prior to their inclusion in the study. After study enrollment, all the patients received oral hygiene instructions to ensure adoption of the correct toothbrushing technique. Clinical Trial Registration:ChiCTR1900022575.

The inclusion criteria were as follows:

- Residual KMW at the buccal site of implant< 2 mm in the mandibular posterior region
- No previous soft tissue augmentation procedure at the experimental site
- At least 18 years old
- Good oral hygiene defined as a full-mouth plaque score ≤ 25% [21]
- Self-reported smoking of ≤ 10 cigarettes/day
- Having signed an informed consent form
  The exclusion criteria were as follows:
- Allergy to collagen
- Diseases affecting connective tissue metabolism
- Untreated periodontal disease
- Unable to maintain oral hygiene
- Systemic diseases or pregnancy

### Allocation

Patients were assigned to the test group (XCM) or control group (FGG) based on their KMW at the buccal site of the implant. Patients with residual KMW ≤ 0.5 were treated with FGG; otherwise, they were treated using XCM or FGG while considering an even number of patients in each group.

### Surgical procedures and postoperative care

Three surgeons (YL/YZ/YW) with more than 10-year experience performed the surgeries. Standardized surgical methods were used by all surgeons. After local infiltration anesthesia using Primacaine Adrenaline (Produits Dentaires Pierre Rolland, Acteon Pharma Division, Merignac, France), a split-flap was prepared using a #15 stainless steel blade at the alveolar ridge crest in the buccal site of implant. The split-flap was apically sutured using resorbable sutures (4/0 Vicryl) and immobilized. The previously inserted implants were uncovered, followed by the insertion of healing abutments. The mesiodistal distance of the recipient bed was measured. In the control group, a graft with matching length, a width of 6~8 mm, and intermediate thickness (about 1 mm) was harvested from the palate between the maxillary canine and first molar (Fig. 5b). The graft was sutured to the recipient site using resorbable sutures (4/0 Vicryl) and immobilized. The donor site was sutured using an iodoform sponge as a wound dressing. In the test group, an XCM (Mucograft®, Geistlich Pharma AG, Wolhusen, Switzerland) piece of appropriate size was selected (15 mm × 20 mm or 20 mm × 30 mm). XCM was trimmed according to the dimension of the recipient bed, with a matching length and width, and then applied in a dry state. XCM was sutured to the recipient bed using resorbable sutures (4/0 Vicryl) with the spongious layer to the periosteum (Fig. 5f). The surgical site was left exposed for healing. Ibuprofen (300 mg) was prescribed at 6 h postoperatively and subsequently taken when necessary. Chlorhexidine solution (0.2%) was administered as anti-infective therapy for 2 weeks. During the 2 postoperative weeks, the patients were instructed to resume toothbrushing.

### PROMs

One investigator, not among the surgeons who performed the surgeries, performed the PROMs.

### Intraoperative PROMs

Immediately after surgery, patients were asked to employ a 100-mm VAS to answer the following five questions: 1. How much pain did you feel during the operation? 2. How much stress did you feel during the operation? 3. How much nausea did you feel during the operation? 4. How was your tolerance to the time of the surgery? and 5. How was your acceptance of the surgical procedure? “Most satisfied feeling” and “most unsatisfied feeling” were indicated as extremes on the 100-mm lines. Moreover, the patients were asked to give the reason when the score for the fifth question exceeded 50 mm.

#### PROMs during the early healing stages

A questionnaire based on VAS scales was postoperatively administered to the patients. The questionnaire assessed daily pain, swelling, and bleeding from postoperative day 1 to 14. Pain perception was further recorded with VAS during daily oral activities, including speaking, chewing, drinking, and oral hygiene care. In the FGG group, pain perception was compared between donor site and recipient site. On this scale, 0 and 100 mm represented “absence of discomfort” and “severe discomfort,” respectively. Additionally, the number of analgesics (Ibuprofen) consumed was recorded during the early healing stage. The following questions were included in the questionnaire: 1. How much pain do you feel today? (from day 1 to day 14); 2. How much swelling do you feel today? (from day 1 to day 14); 3. How much bleeding do you feel today? (from day 1 to day 14); 4. How many pain killers did you consume today? (from day 1 to day 14); 5. How much pain did you feel when speaking?; 6. How much pain did you feel when chewing?; 7. How much pain did you feel when drinking?; 8. How much pain did you feel during oral hygiene care?; and 9. Considering the pain perception you felt, which one was more severe? (A. donor site B. recipient site C. equal).

#### PROMs after 6-month follow-up

At the 6-month follow-up, the willingness for retreatment was investigated using the following question: “Would you be willing to undergo the same procedure again, if necessary?” (yes/no), when the answer was “no,” the patient was asked to give the reason. VAS scales were used to assess brushing comfort, color satisfaction, and shape satisfaction with the following questions: 1. How much brushing comfort did you feel? 2. How much satisfaction did you feel with the color of the grafted region? 3. How much satisfaction did you feel with the shape of the grafted region? On this scale, 0 and 100 mm represented “extremely dissatisfied” and “extremely satisfied,” respectively.

### KMW and other peri-implant parameters

KMW was measured from the free mucosal margin to the mucogingival junction at the buccal midpoint of implant abutment or crown with a UNC-15 probe, as mentioned in the previously reported methods [4, 11]. KMW was recorded before surgery, immediately after surgery, 3 months after surgery, and 6 months after surgery. The modified plaque index (mPLI), the probing depth (PD), and the modified bleeding index (mBI) were recorded based on the method reported by Mombelli [22]. The gingival index (GI) was measured following the index system reported by LÖE [23].

### Sample size

Based on the sample size calculation for maximum pain level, α = 0.05, power of 80%, standard deviation = 1.28 [17], and minimum clinically significant difference in VAS (δ) of 1, we determined a sample size of 20 patients per group.

### Statistical analysis

Each patient was considered a statistical unit. Descriptive statistics included the mean, standard deviation, median, and quartiles. Pain perception was the primary outcome variable. To comprehensively represent pain, VAS scores were divided into mild (0 ≤ VAS ≤ 30), moderate (40 ≤ VAS ≤ 60), and severe (70 ≤ VAS ≤ 100). The Kolmogorov-Smirnov test was used to assess the distribution normality. Independent sample *T* tests and Mann-Whitney *U* tests were used for normally and non-normally distributed data, respectively. Comparisons of the willingness for retreatment and numbers of ibuprofen taken were performed using the chi-square test. *p* ≤ 0.05 was considered significant. Statistical analyses were performed using the SPSS software (SPSS version 20; SPSS).

## Results

### Patient groups

**Fig. 1:**
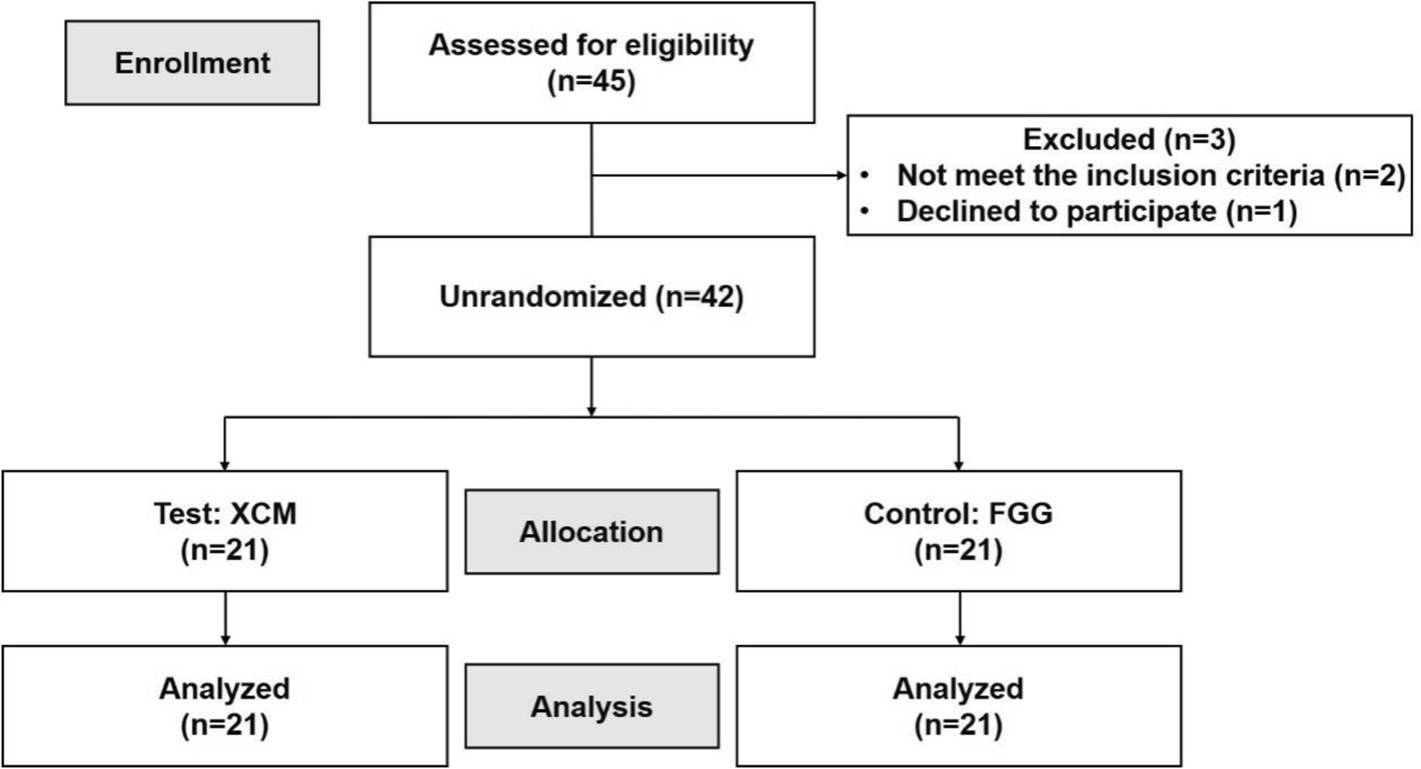
CONSORT flowchart

This study enrolled 42 patients with 21 patients per group; among them, forty-six implants (premolar: 19, molar: 27) and 44 implants (premolar: 12, molar: 32) were allocated to the test and control groups, respectively (Fig. 1). All 42 patients underwent the procedure during the second-stage surgery. In the control group, there were 14 females and 7 males (average age: 45.5 ± 12.3 years; range: 26–65 years). In the test group, there were 13 females and 8 males (average age: 41.0 ± 11.0 years; range: 21–59 years). Two patients in each group reported a history of smoking. The number of individuals with residual buccal KMW ≤ 0.5 mm was 3. Table 1 presents the demographic characteristics of the patients. Baseline data, including gender, age, location of implants, and surgeons, showed no significant between-group differences, indicating good between-group comparability.

**Table 1:** Baseline demographic data

		XCM	FGG	***p***-value
**Age (years)**		41.0 (11.0)	45.5 (12.3)	0.217
**Gender**	Female/Male	13/8	14/7	0.747
**Smoke**	Yes/No	2/19	2/19	1
**Diabetes**	Yes/No	0/21	1/20	1
**Surgeons**	YL/YZ/YW	6/10/5	9/5/7	0.273
**Location of implant**	Premolar/Molar	19/27	12/32	0.161

### PROMs

#### Intraoperative PROMs

There were no statistically significant between-group differences in pain, stress, nausea, and tolerance to time. Outcomes were presented as medians and interquartile ranges. The test group reported higher acceptance to surgery than the control group (0, 0–30.0 vs. 30, 0–50.0; *p* = 0.025) (Table 2). In the FGG group, 8 patients had a VAS of ≥ 50 for acceptance to surgery; among them, 7 patients attributed it to the anxiety of postoperative pain, while 1 patient complained about the surgical time. In the XCM group, 2 patients had a VAS score of ≥ 50 in acceptance to surgery due to intense intraoperative stress and the surgical time being too long, respectively.

**Table 2:** PROMs during the operation

	XCM			FGG			***p***-value
	Median	25%	75%	Median	25%	75%	
**Pain**	10.0	0	35.0	20.0	10.0	32.5	0.156
**Stress**	20.0	5.0	65.0	30.0	10.0	55.0	0.869
**Nausea**	0	0	10.0	0	0	25.0	0.347
**Tolerance to time**	40.0	10.0	60.0	50.0	30.0	50.0	0.758
**Acceptance to surgery**	0	0	30.0	30.0	0	50.0	0.025*

Median, first, and third quartile of the PROMs on a VAS from 0 to 100 mm. A Mann-Whitney *U* test was used to calculate significance levels (*p*-value). *Statistically significant difference between groups (*p* < 0.05)

#### PROMs during the early healing stages

**Fig. 2:**
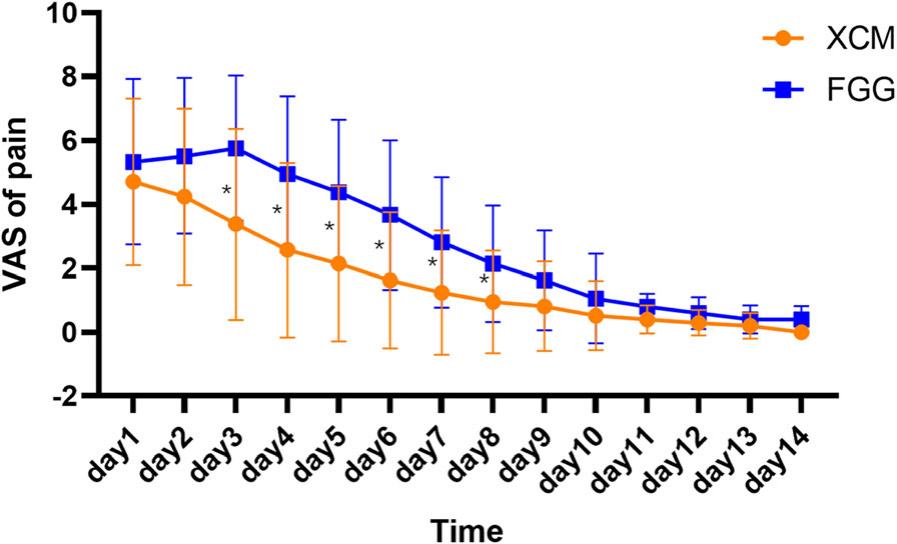
Patients reported pain perception during the early healing stages. *Statistically significant difference between groups (*p* < 0.05)

Table 3 shows the 2-week follow-up results. The maximum pain severity was significantly different between the control (61.9 ± 20.2) and test groups (46.7 ± 25.9; *p* = 0.040). The pain duration in the test group (5.52 ± 3.57) was significantly shorter than that in the control group (8.48 ± 2.80; *p* = 0.005). The extended pain duration was approximately 3 days. There was no between-group difference in the duration of mild and severe pain; however, the duration of moderate pain was significantly higher in the control group than in the test group. Regarding the change in daily pain perception during the 2-week follow-up, the pain level continuously declined in the test group; conversely, in the control group, it continuously increased during the first 3 days and decreased subsequently (Fig. 2). The higher pain level in the control group mainly extended from day 3 to 8. In the control group, 16 and 4 patients reported higher pain at the donor and recipient sites, respectively; moreover, 1 patient reported equal pain intensity at both sites at the 2-week follow-up. There were significant between-group differences with regard to pain perception during chewing (XCM: 47.1 ± 26.9, FGG: 62.9 ± 20.3, *p* = 0.039) and speaking (XCM: 25.2 ± 16.3, FGG: 41.4 ± 21.3, *p* = 0.009).

**Table 3:** PROMs during the early healing stages

	XCM	FGG	***p***-value
**Duration of pain (day)**	5.52 ± 3.57	8.48 ± 2.80	0.005*
Mild pain (day)	2.71 ± 2.17	2.90 ± 2.32	0.785
Moderate pain (day)	2.10 ± 2.32	3.90 ± 2.86	0.030*
Severe pain (day)	0.81 ± 1.66	1.52 ± 2.14	0.234
**Maximum pain**	46.7 ± 25.9	61.9 ± 20.2	0.040*
**Pain during daily oral activities**			
Speaking	25.2 ± 16.3	41.4 ± 21.3	0.009*
Chewing	47.1 ± 26.9	62.9 ± 20.3	0.039*
Drinking^a^	0 (0)	0 (10.0)	0.259
Oral hygiene care^a^	10.0 (20.0)	10.0 (20.0)	0.275
**Duration of swelling (day)**	6.76 ± 3.05	8.00 ± 2.43	0.153
**Maximum swelling**	56.2 ± 26.0	63.8 ± 23.0	0.329
**Duration of bleeding (day)**	2.48 ± 2.69	2.86 ± 2.43	0.633
**Maximum bleeding**	26.2 ± 25.2	36.7 ± 24.6	0.180

^a^Data are presented as median (interquartile range), and Mann-Whitney *U* tests were used to calculate significance levels (*p*-value). Other data are presented as mean ± standard deviation. Independent sample *T* tests were used to calculate significance levels (*p*-value). *Statistically significant difference between groups (*p* < 0.05)

**Fig. 3:**
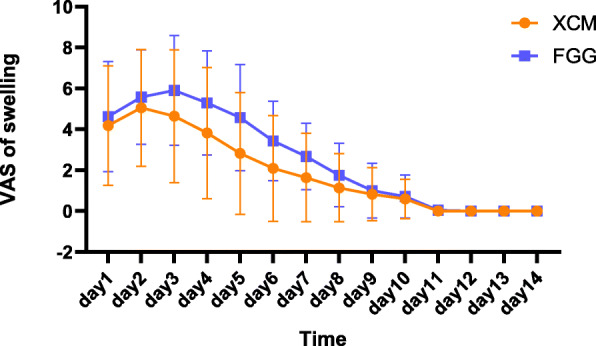
Patients reported swelling perception during the early healing stages

**Fig. 4:**
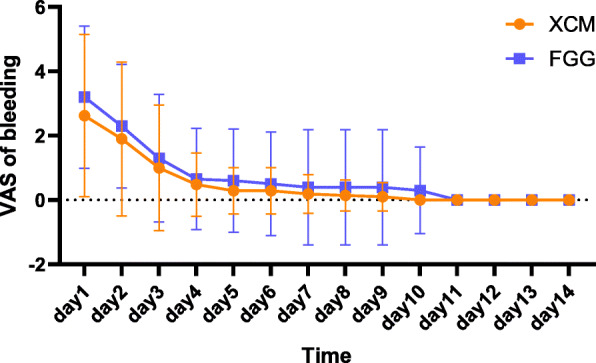
Patients reported bleeding perception during the early healing stages

The bleeding and swelling related to the surgery were significantly experienced during the first 3 postoperative days (Figs. 3 and 4). The swelling and bleeding in the control group were higher, but no statistically significant between-group differences were shown (Table 3). The analgesic consumption was similar (Table 4).

**Table 4:** Numbers of ibuprofen taken during the early healing stages

Numbers of ibuprofen taken	XCM	FGG	Total	***p***-value
**0**	11	11	22	
**1~5**	7	5	12	
**6~10**	1	4	5	
**> 10**	2	1	3	
**Total**	21	21	42	0.481

Chi-square test was used to calculate significance level (*p*-value)

#### PROMs after 6-month follow-up

There was a statistically significant between-group difference in the willingness for retreatment in the test (yes: 16/21 [76%]) and control group (yes: 9/21 [43%]), *p* = 0.021) (Table 5). The reasons for the unwilling answers were as follows. In the FGG group, 9 patients attributed it to the postoperative pain experience, while 3 patients complained about the chew disability. In the XCM group, 3 patients decried the postoperative pain experience, 1 patient complained of swelling, and 1 patient said the benefit of the surgery was not fully felt. There were no significant between-group differences in brushing comfort, color satisfaction, and shape satisfaction.

**Table 5:** PROMs after 6-month follow-up

	XCM	FGG	***p***-value
**Comfort of brushing**^a^	8.17 ± 2.05	7.71 ± 2.39	0.514
**Satisfaction of color**^a^	8.45 ± 1.82	7.71 ± 1.99	0.251
**Satisfaction of shape**^a^	8.34 ± 1.62	7.61 ± 2.20	0.255
**Willingness to retreat**^b^			
Yes	16	9	0.021*
No	5	12	

Data are presented as mean ± standard deviation except for willingness to retreat. *Statistically significant difference between groups (*p* < 0.05). ^a^Independent sample *T* tests. ^b^Chi-square test

### KMW and other peri-implant parameters

**Fig. 5:**
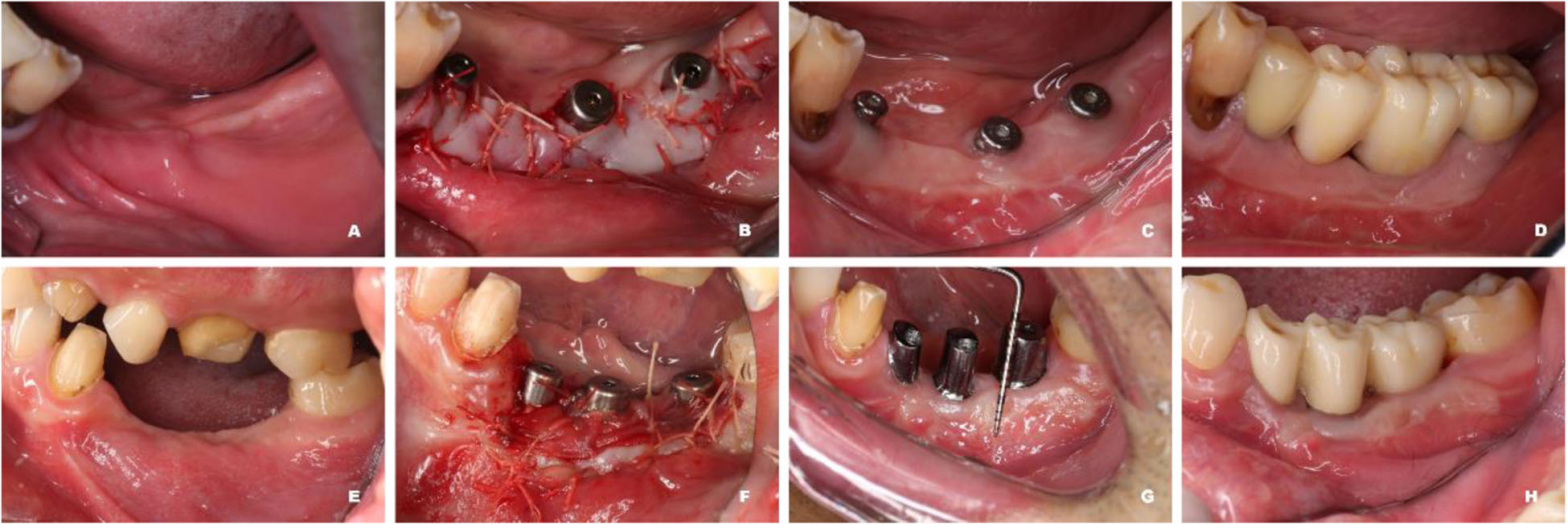
Representative cases in both groups. **a**–**d** Representative case in FGG group, **a** before surgery, **b** immediately after surgery, **c** 3 months after surgery, **d** 6 months after surgery. **e**–**h** Representative case in XCM group, **e** before surgery, **f** immediately after surgery, **g** 3 months after surgery, **h** 6 months after surgery

These outcomes are shown in Table 6. There was no significant between-group difference in the KMW before surgery and 3 months after surgery. The KMW at the 6-month follow-up was significantly lower in the XCM group (XCM: 2.82 ± 1.58 mm, FGG: 3.74 ± 1.76 mm, *p*=0.010). The shrinkage rate of KMW was significantly higher in the XCM group during the 6-month follow-up (XCM: 65 ± 21%, FGG: 47 ± 23%, *p*=0.000). The gain of KMW was significantly higher in FGG than in XCM (XCM: 1.57 ± 1.69 mm, FGG: 2.68 ± 1.80 mm, *p* = 0.003). Other peri-implant parameters showed no significant between-group differences. Two representative cases from each group are shown in Fig. 5.

**Table 6:** Changes of KMW with time and other peri-implant parameters at 6-month follow-up

		XCM (n = 46)	FGG (n = 44)	***p***-value
**KMW**^a^ **(mm)**^b^	Baseline	1.25 ± 0.53	1.07 ± 0.50	0.092
	Immediately after surgery	8.72 ± 3.56	7.01 ± 1.77	0. 005^*^
	3 months after surgery	3.63 ± 1.79	4.22 ± 1.91	0.137
	6 months after surgery	2.82 ± 1.58	3.74 ± 1.76	0.010^*^
**Shrinkage rate of KMW at 6 months (%)**^b^		65 ± 21	47 ± 23	0.000^*^
**Gain of KMW at 6 months (mm)**^b^		1.57 ± 1.69	2.68 ± 1.80	0.003^*^
**mPLI**^c^		0 (0)	0 (0)	0.570
**GI**^c^		0 (0)	0 (0)	0.506
**PD**^b^		2.70 ± 0.71	2.66 ± 0.89	0.796
**mBI**^c^		0 (0)	0 (0)	0.728

^a^Keratinized mucosa width. ^b^Data are presented as mean ± standard deviation. Independent sample *T* tests were used to calculate significance levels (*p*-value). *Statistically significant difference between groups (*p*<0.05). ^c^Data are presented as median (interquartile range), and Mann-Whitney *U* tests were used to calculate significance levels (*p*-value)

## Discussion

This study compared the PROMs and clinical outcomes between XCM and FGG within a 6-month follow-up period. We found that compared with FGG, XCM demonstrated higher surgical acceptance, less postoperative morbidity, especially for pain perception, greater willingness for retreatment, but less KMW augmentation in the posterior mandible.

Compared with the gold standard FGG, XCM has been extensively shown to be effective in treating peri-implant keratinized mucosa defects in suitable cases [9–11, 13]. However, the present study showed that the gain of KMW in the XCM group was inferior to the FGG group. Our study is consistent with the study by Lim, which showed more increase of KMW in FGG than in XCM in the posterior mandible [4]. The reason for the difference could be that the present study only included cases in the mandibular posterior region, which was near the external oblique ridge and was frequently accompanied with high muscle attachment and shallow vestibule depth. Therefore, the shrinkage of XCM was more significant than that of FGG. Nevertheless, the mean KMW at 6 months was > 2mm in the XCM group, and other peri-implant parameters did not show significant between-group difference. This indicated that KMW could be increased to an adequate amount (≥ 2mm) with XCM, although not optimal, and not influence the peri-implant soft tissue health in the 6-month follow-up.

XCM was associated with higher surgical acceptance but not less intraoperative discomfort in terms of pain, stress, nausea, and tolerance to time intraoperatively. This was consistent with a previous report suggesting that XCM conferred a significantly lower hardship perception than connective tissue graft for buccal soft tissue augmentation at the implant site; however, there was no statistically significant difference for perceived pain [12]. Additionally, McGuire et al. reported that patients preferred the XCM procedure [17]. These findings indicate that XCM is more preferred by patients. In the FGG group, 8 patients reported VAS ≥ 50 in terms of surgical acceptance. Among them, 7 patients attributed it to the anxiety of postoperative pain. This result indicated that the anxiety for postoperative pain during the early healing stage could have resulted in lower surgical acceptance in the FGG group.

Compared with XCM, FGG was associated with higher severity and duration of pain perception following keratinized mucosa augmentation during the early healing stages. This was consistent with previous reports, which suggested that XCM was associated with lower pain intensity and duration than autogenous soft tissue graft during the early healing period [10, 12]. Notably, in our study, the duration of moderate pain was shorter in the XCM group; however, there was no between-group difference in mild and severe pain. These findings may allow surgeons to better inform patients about postoperative pain for the patient’s decision-making.

Regarding the daily pain perception change during the 2-week follow-up, there was a between-group difference in the trend of pain change (Fig. 2). The pain level continuously declined in the test group; conversely, it increased during the first 3 days and decreased subsequently in the control group. This is consistent with the report by McGuire et al., which suggested that FGG conferred a high pain level during the first few days [17]. The different trends of pain change may be explained in terms of the healing process of mucosal wounds. At the recipient site, the initial healing phase of up to 3 days involves the survival of the grafted tissue through plasmatic circulation from the recipient bed [24]. An exudate layer or a blood clot may be established to protect the wound area; this is followed by the establishment of blood clot perfusion. Pain perception is continuously decreased during this phase and reaches a very low level [24]. However, in the FGG group, the higher pain level during the initial phase could be attributed to the healing process at the donor site proceeding by secondary intention. Furthermore, we found that the pain perception during daily oral activities, including chewing and speaking, was significantly higher for FGG. In the FGG group, the palatal wound site was associated with higher pain (16/21) than the recipient site. Previous studies have suggested that mechanical stress and periosteal stretching are major sources of pain [25]. Complete epithelialization of the palatal donor site takes approximately 2–4 weeks [26]. The denuded donor site is more susceptible to external mechanical stimuli during the initial healing stage. Therefore, local stimulus of the palatal donor site, including mastication and pronunciation, may amplify the postoperative pain.

Pain was the main postoperative symptom in both procedures in this study. Pain management is a fundamental human right and an important part of dental care [27, 28]. However, international consensus on pain management for dental soft tissue graft surgery has not been reached [29]. Regarding systemic measures, incomplete medicine was prescribed. The most common postoperative instruction has been to use analgesics when necessary or no specific medications are recommended [9, 12, 14], which is consistent with our study. In the present study, anxiety related to postoperative pain and pain experienced during the early healing stage were the main complaints. Therefore, specific measures for minimizing postoperative patient pain perception are demanding to allow more patient’s tolerance to soft tissue graft procedures.

The bleeding and swelling related to the surgery were considerable during the first 3 postoperative days. Compared with XCM, FGG caused non-significantly greater bleeding and swelling levels, which is inconsistent with previous findings. Maiorana et al. reported that XCM was a great hemostatic with no patient-reported bleeding during the postoperative period [30]. Furthermore, Wang et al. found that XCM resulted in much less postoperative bleeding than FGG [13]. Our findings suggest that proper surgical procedures could result in similar bleeding and swelling levels for FGG and XCM procedures.

Compared with FGG (43%), XCM was associated with a higher patients’ willingness for retreatment (76%). Patients mainly attributed it to the pain experience for FGG. However, there was no between-group difference in satisfaction with the treatment outcomes. Most patients favor less traumatic procedures [31]. A recent study found that having undergone autogenous soft tissue grafting influenced the patients’ decision to undergo future treatment [32]. The less traumatic procedure and postoperative morbidity associated with XCM may have contributed to the higher willingness to retreat.

Above all, XCM provided more positive patient experiences than FGG following peri-implant keratinized mucosa augmentation, but XCM was associated with less KMW augmentation than FGG in the posterior mandible. This study provided comprehensive information in terms of PROMs in the practice of informed consent. From the aspect of clinical relevance, in the posterior mandible, XCM may be indicated when patients can bear little pain.

This study has several limitations. First, the use of multiple surgeons and graft dimensions compromised consistency. Second, the allocation of groups was not randomized. Nonetheless, the selected study population was well reflective of the PROMs. Future multi-center randomized clinical trials with higher intergroup consistency are needed to evaluate the PROMs following soft tissue grafts.

## Conclusions

Within the limitation of the present nonrandomized study, XCM demonstrated more positive PROMs than FGG during different postoperative phases, mainly for less pain perception during the early healing stage, but was inferior to FGG in terms of gain of KMW. For KMW augmentation in the posterior mandible, XCM may be indicated when patients can bear little pain.

## Data Availability

Data sharing is not applicable to this article as no datasets were generated or analyzed during the current study.

## Abbreviations

PROMs: Patient-reported outcome measures
XCM: Xenogeneic collagen matrix
FGG: Free gingival graft
KMW: Keratinized mucosa width
VAS: Visual analog scales
